# The social-economic impact of COVID-19 pandemic: implications for potential forest degradation

**DOI:** 10.1016/j.heliyon.2020.e05354

**Published:** 2020-10-26

**Authors:** Golar Golar, Adam Malik, Hasriani Muis, Achmad Herman, Nurudin Nurudin, Lukman Lukman

**Affiliations:** a Forestry Faculty of Tadulako University, Central Sulawesi, Indonesia; b Social and Politic Faculty of Tadulako University, Central Sulawesi, Indonesia; c Forest Management Unit (FMU) Toili Baturube, Central Sulawesi, Indonesia; d Forest Management Unit (FMU) Sintuwu Maroso, Central Sulawesi, Indonesia

**Keywords:** Environmental science, COVID-19 pandemic, Social-economic impact, FMU, Forest degradation

## Abstract

This article presents an analysis of the potential forest damage that occurred due to the COVID-19 pandemic in rural communities on the Forest Management Unit (FMUs). It focused on forest utilization and deforestation before and during the epidemic. Base on The data on online surveys using Google form instruments, Zoom meetings, and in-depth telephone interviews with the informants. The data of the research were analyzed descriptively using the mind mapping method. The data analysis shows that social and economic impacts potentially enhance the threat of forest resource utilization–increasing pressure on the forest due to the increase in forest product demand. Even though the government made efforts to minimize forest degradation and prevent illegal logging, the communities didn't follow the policy because there were no alternative solutions. The timber logging is carried out into a threat to forest degradation when it's not immediately prevented. The FMU needs to improve access to rural living near the forest to increase their forest income. These solutions are crucial for reducing illegal logging activities and forest degradation in the pandemic.

## 1 Introduction

**Figure 1:**
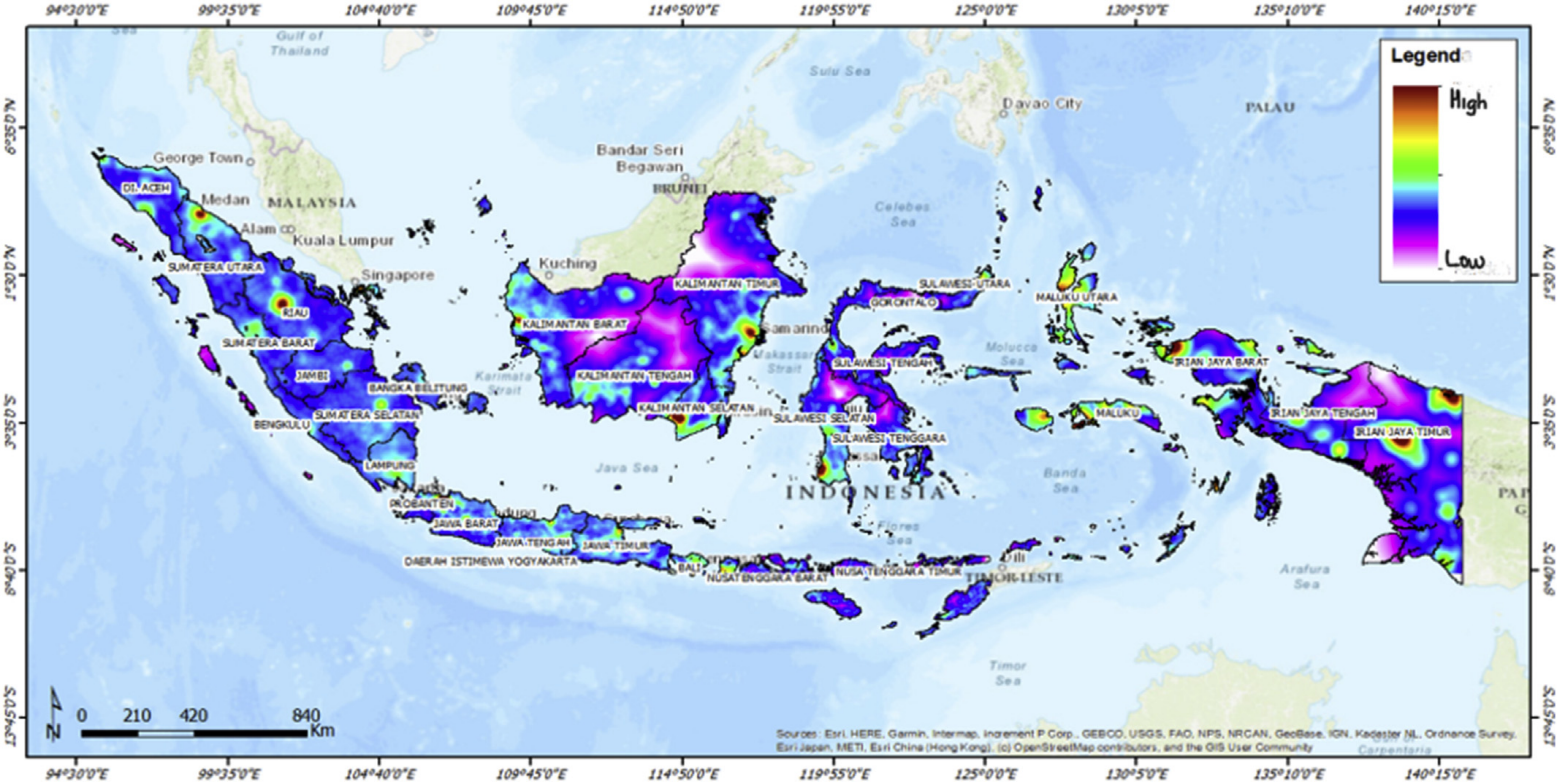
Cities with the extensive COVID-19 in Indonesia Source: https://covid19.lapan.go.idhttps://infeksiemerging.kemkes.go.id (access: 20 Oktober 2020).

The COVID-19 pandemic has had a global impact on hitting more than 200 countries [1]. Indonesia is one of the countries where regencies and cities have been most struck [2], with 370 towns and districts affected (see Figure 1). The number of victims infected until October 2020 is as many as 403.523 people, 13.654 deaths, and 329.339 people recovered https://infeksiemerging.kemkes.go.id (https://covid19.lapan.go.id). Besides the health issues, the COVID-19 pandemic has also significantly impacted social and economic aspects in the third and established countries [3]. The global economy slowed down as businesses were affected by the World Health Organization (WHO), which classified the outbreak as a pandemic [4].

This situation exacerbates some countries' policies in responding to this pandemic, from social distancing to lockdown policies [5]. In Indonesia, the impact of the COVID-19, especially for the national economy, occurs almost in all lines. Applying a large-scale social restriction policy (PSBB) affects the suspension of production activities from large-scale industries to household-scale enterprises [2].

Social shock appears when companies are forced to reduce their labor to save their costs [6]. Moreover, those who cannot survive and fulfill their life needs in the COVID-19 will go back to their hometown [7]. One of the reasons they chose to return to their hometown is that the price of goods is steadily increasing while purchasing it is decreasing [8]. In Indonesia, since the government issued social distancing policies through a large-scale social restriction program, the returned urban population's percentage continues since the beginning of March 2020 [2].

The poverty rate in Central Sulawesi reached 402,023 people, or the proportion reached 13.18%. COVID-19 has pushed the poverty rate up to 14.13%, from 402,023 people to 458.8450 due to the impact of the termination of employment. The jobs directly affected are the agricultural, forestry, fisheries, major trade, and retail sectors. Moreover, for those who live in rural areas with limited resources (https://metrosulawesi.id/2020/04/12dampak-covid-19-pada-perekonomian-sulteng/).

The new problems that arose in the countryside are the comeback population from the cities, the threats of transmission of the COVID-19 virus, and the villages' social and economic aspects [9, 10]. Those who choose to return to the village due to employment termination suffered an additional burden with having a lack of natural resources. Coupled with the villagers compete to utilize natural resources in fulfilling their life needs in the pandemic [6, 8].

The study conducted by [11] reported that natural resources, especially forests, are often used as objects of utilization by rural communities. They harvest timber, fuelwood, non-timber forest products, mining, land use, and environmental services [12, 13]. Likewise, some previous studies have proved that the utilization of natural resources in rural often affects the destruction of land and forests [14, 15].

However, it is not easy to know the pandemic's environmental impact because the damage caused by human intervention takes a long time. We can learn from trends that occur in the field, especially in forest resources [10, 16, 17].

This paper presents the potential for deforestation and land degradation due to COVID-19 pandemic in rural areas near the forests in Central Sulawesi. The analysis focused on forest deforestation and land-use change issue during the pandemic and found out the efforts made by each FMUs in tackling the socioeconomic impact of the epidemic. This information can use as a reference for the government in establishing environmental damage mitigation policies for the COVID-19 pandemic.

## 2 Research methods

### 2.1 Study area

**Figure 2:**
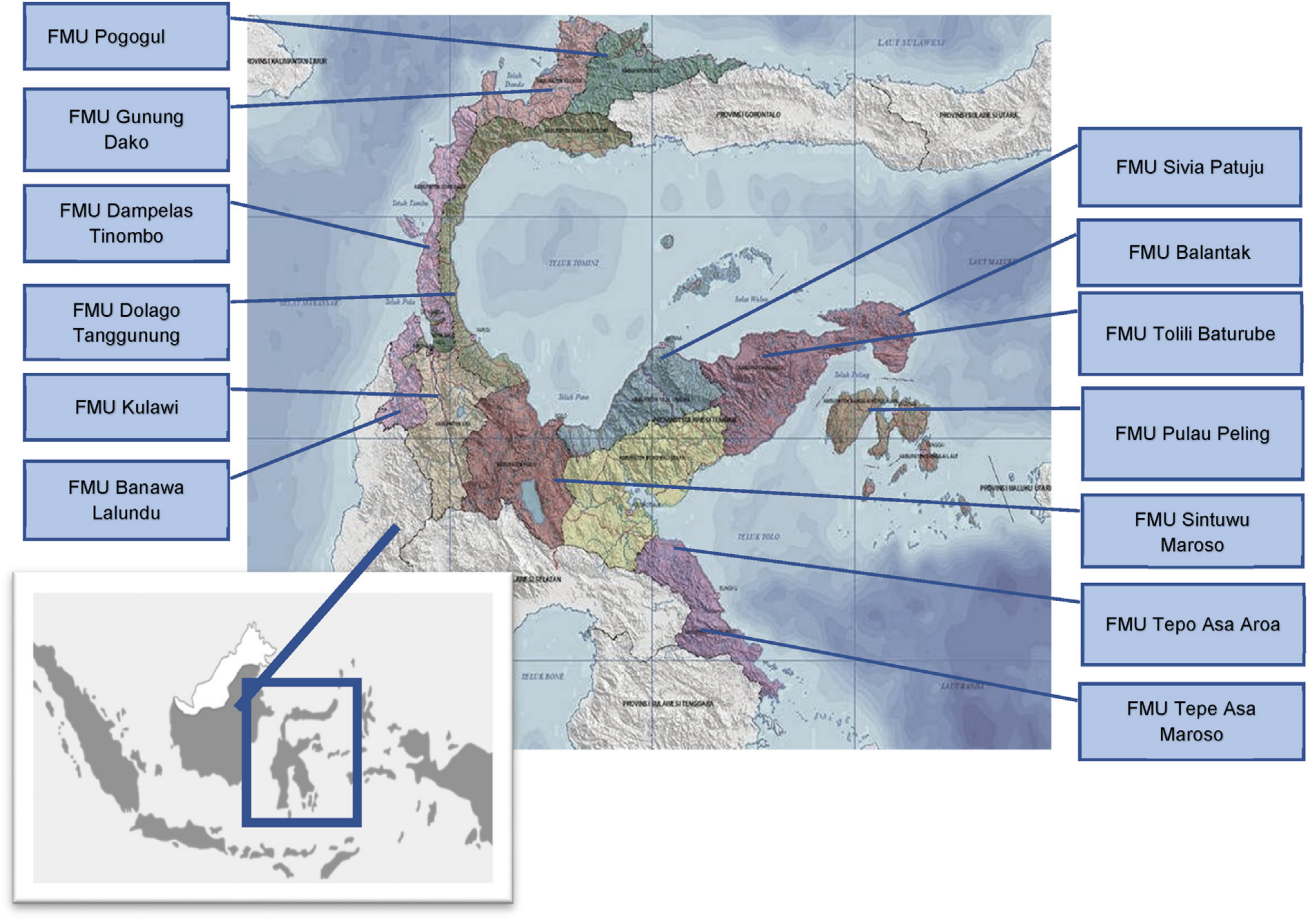
FMUs location in Central Sulawesi, Indonesia (Map *source:*https://maps.googles.com).

Central Sulawesi has a forest area of 4,410,293.84 ha or 72.22% of the total province area. Currently, the entire forest area distributes into the managed space of the FMUs. There are 13 FMUs in Central Sulawesi Province, including Dampelas Tinombo, Sivia Patuju, Toili Batu Rube, Pulau Peling, Banawa Lalundu, Pogogul, Dolago Tanggunung, Kulawi, Sintuwu Maroso, Tepo Asa Aroa, Tepo Asa Maroso, Gunung Dako, and Balantak. Our research took place in all FMUs in Central Sulawesi province (see Figure 2).

The entire FMU regions have state forest status. Based on the type and function of the forest area in each FMU consists of production forest (HP), protection forest (HL), and conservation forest (HK). FMU management in Central Sulawesi only focused on HP and HL. Both are under the direction of the Central Sulawesi Provincial Forestry Service.

FMU's primary duties and functions are to conduct forest management, which includes: forest management and the preparation of forest management plans, forest utilization; use of forest areas; forest rehabilitation and reclamation; and Forest protection and nature conservation. FMU has an essential mission in carrying out the community empowerment facilities presenting information on potential forestry development investment opportunities in its managed areas.

The strategic roles of FMU are: optimizing people's access to forests is one of the ways for conflict resolution, optimizing potential (timber, non-timber, environmental services) by following the conditions at the field level Increasing successful forest rehabilitation and reclamation. However, they direct the utilization of non-timber forest products and environmental services, shown in Table 1. In the FMU region, there are groups of forest farmers who are built by FMUs. The group will be empowering in the utilization of non-timber forest products.

**Table 1:** Characteristics and utilization of forests in the FMU's region.

FMU	Total (Ha)	Types of Utilization			
		Number of Farmer Group	Non-Timber	Environmental Services	Land Use
Toili Baturube	250.778	37	- Rattan - Resin - Honey Bee - Palm Sugar	Ecotourism	Forest Community
Pulau Peling	117.000	5	- Rattan - Honey Bee	none	none
Banawa Lalundu	110.000	35	- Rattan - Honey Bee	Ecotourism	none
Pogogul	199.524	24	- Rattan - Resin - Honey Bee	none	none
Dolago Tang-gunung	239.288,23	17	- Rattan - Resin - Honey Bee	Ecotourism	Coffee tree Durian Tree
Kulawi	219.418,78	37	- Rattan - Honey Bee - Pinus	none	none
Sintuwu Maroso	322.457,84	149	- Rattan - Resin - Honey Bee	Ecotourism	Mushroom cultivation
Tepo Asa Aroa	327.614,22	23	- Rattan - Resin - Honey Bee	none	none
Dampelas Tinombo	240.000	15	- Rattan - Resin - Honey Bee - Palm Sugar	none	Rubber tree (near the forest)
Sivia Patuju	388.299,89	57	- Rattan - Resin - Honey Bee - Palm Sugar	none	none
Tepe Asa Maroso	190.956	17	- Rattan - Resin - Honey Bee - Palm Sugar	none	none
Gunung Dako	171.292,51	138	- Rattan - Resin - Honey Bee - Palm Sugar	none	none
Balantak	289.289,72	28	- Rattan - Resin - Honey Bee - Palm Sugar	none	Candlenut Cultivation

### 2.2 Data collection

This research's data collection methods include online surveys using Google form instruments, Zoom meetings, and in-depth interviews using a mobile phone. It distributed a questionnaire of Google form to all heads of FMU in Central Sulawesi (https://forms.gle/ngUGP3omUpxEr87w7). Once the data from the Google form is collected, all informants (13 people) are invited to meet virtually with the research team through Zoom meetings to confirm and extract additional information from the heads of FMU. This method is more effective than the conventional method because it's more flexible in presenting all informants in a virtual meeting room without meeting.

This study uses two FMUs as a sample for extracting information about the impact of forest damage to the Sintuwu Maroso and Dampelas Tinombo, because they found the illegal logging activity during the pandemic in both locations. Collecting data methods is shown in Table 2.

**Table 2:** Collecting data method.

No	Data	Method	Informants
1	1. Social and economic impacts 2. Forest utilizing 3. The efforts of FMUs in suppressing the COVID-19 4. Land-use changes	- Google form - Zoom meetings	FMUs Head (13 people)
2	The potential of Forest Damages (Illegal logging and deforestation)	Depth interview by call (Using mobile phone)	1. FMUs Head (2 people) 2. Forest Rangers (4 People) 3. Representation farmer's group (5 people)

### 2.3 Data analysis

The research data were analyzed qualitative and descriptive using the Mind mapping method [18], with the MindMaple Pro version 1.3.1 software. This method reveals the causal relationship between pandemic to the community's socioeconomic impacts and the potential forest damage. Deforestation data were analyzed descriptively using the current conditions on FMU (Sintuwu Maroso and Dampelas Tinombo) before and during the pandemic.

Coupled with another user to find out the efforts made by each FMU in tackling the socioeconomic impact of the pandemic on the potential for deforestation is descriptively analyzed based on the results of online questionnaires and depth interviews with the informants.

## 3 Result and discussion

### 3.1 Result

#### 3.1.1 Social and economic impact

The social and economic impacts on rural areas around FMU are grouped based on the evolving issues: social interaction, community income, limited resources, government policy, the role of FMU in addressing the impact of pandemic (see Table 3). They were collecting data from the informants (FMU heads).

**Table 3:** The Main Issue related to Socio-economic Impact of People in FMU due to COVID-19.

No	FMU	The Main Issue	The conditions in the field
1	Toilil Baturube	1. The decrease in Social interactions 2. People's income 3. FMU role	- Low interactions with each other - Suspicion each other - Picking up non-timber forest products - Clearing land in forested areas - No alternative empowerment program
2	Pulau Peling	1. Limited resources 2. Government policy 3. FMU role	- Farming activity stalled - Logging hunting - Grand from the government is less right target - Temporary suspension of some empowerment programs - The intensity of FMU officer visits decreases during pandemic-19
3	Banawa Lalundu	1. People's income 2. Government policy	- Utilization of non-timber forest products - Forest land clearing - Grand from the government is less right target
4	Pogogul	1. Limited resources 2. FMU role	- Competition in the utilization of natural resources - Forest clearing for business land - Temporary suspension of some empowerment programs - The intensity of FMU officer visits decreases during the pandemic
5	Dolago Tang-gunung	1. Limited resources 2. People's income	- No have to land - Clearing of forest land - Collecting non-timber forest products (Rattan and Honey bee) - Utilization of environmental services (Ecotourism)
6	Kulawi	1. Limited resources 2. People's income 3. FMU role	- Farming activity stalled - New land clearing in forest areas - Gardening activities in forested areas - The intensity of FMU officer visits decreases during the pandemic
7	Sintuwu Maroso	1. The decrease in Social interactions 2. People's income 3. Limited resources	- Low interactions with each other - Doing illegal logging activities - Clearing land in forested areas - Collecting non-timber forest products
8	Tepo Asa Aroa	1. Limited resources 2. FMU role	- There is a threat to the utilization of natural resources from other villages - Forest clearing for family business land - Temporary suspension of some empowerment programs - There is already a program planned, but it is not yet running
9	Dampelas Tinombo	1. The decrease in Social interactions 2. People's income 3. Limited resources	- Low interactions between the village community and FMU - Doing illegal logging activities - Clearing land in forested areas - Collecting non-timber forest products (Rattan and Palm)
10	Sivia Patuju	1. Limited resources 2. FMU role	- Limited ownership of community land - Forest clearing for family business land - Temporary suspension of some empowerment programs - KPH visits since pandemic are infrequent
11	Tepe Asa Maroso	1. Limited resources 2. FMU role	- Farming activity stalled - Land ownership limitations - Temporary suspension of some empowerment programs - The intensity of FMU officer visits decreases during the pandemic
12	Gunung Dako	1. Limited resources 2. FMU role	- Farming activity stalled - Logging hunting - Temporary suspension of some empowerment programs
13	Balantak	1. People's income 2. Government policy	- Farming activity stalled - Logging hunting - Temporary suspension of some empowerment programs

#### 3.1.2 The potential forest damage

The utilization of forest by communities has been a long time before the COVID-19 pandemic. that is in the form of timber, non-timber, and planting forest. Nevertheless, there is an increase at this time. Comparative data on forest and land utilization before and after the COVID-19 pandemic is shown in Table 4.

**Table 4:** The trend of land use and forest resources in the region of Sintuwu Maroso and Dampelas Tinombo *(Before and After COVID-19*).

No	FMU	Before COVID-19	During COVID-19	Trend
1	Sintuwu Maroso	1. Community get income from non-timber forest products 2. The community partners with companies in land development 3. Land use, applying agroforestry patterns	1. Timber logging for commercial purpose 2. Suspend Some partnership activities 3. Land clearing in the forest to be used as plantation land	1. Increase timber logging activity 2. Increase the illegal logging case 3. Increase the motivation of land use for commercial purpose 4. Empowerment programs tend not to run well
2	Dampelas Tinombo	1. Timber logging for personal or social needs 2. Community get income from non-timber forest products 3. The empowerment of the land use program of FMU is running well	1. Timber Logging for commercial purpose 2. Land clearing in the forest to be used as plantations land 3. The empowerment of the land use program of FMU is running well	

Table 4 shows an increase in land use clearing, increasing timber logging, and empowerment programs not to run well in both FMU since the pandemic. As compensation, they are trying to find business alternatives that can generate income quickly.

### 3.2 Discussion

#### 3.2.1 The impact of the pandemic on social and economic aspects of society

**Figure 3:**
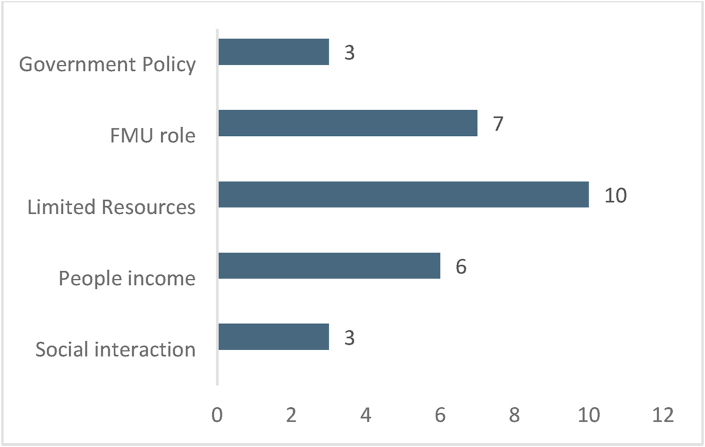
The Dominant Issues for Socioeconomic impact of People in FMU.

The COVID-19 epidemic has made a broad impact on human life globally. In Indonesia, the effect is extensive, especially on the social and economic aspects [2]. Table 3 shows the socioeconomic impact of people in the FMU Region due to the pandemic. The most dominant issue found are limited resources (land and startup capital); FMU's role; and people's incomes (see Figure 3)

**Figure 4:**
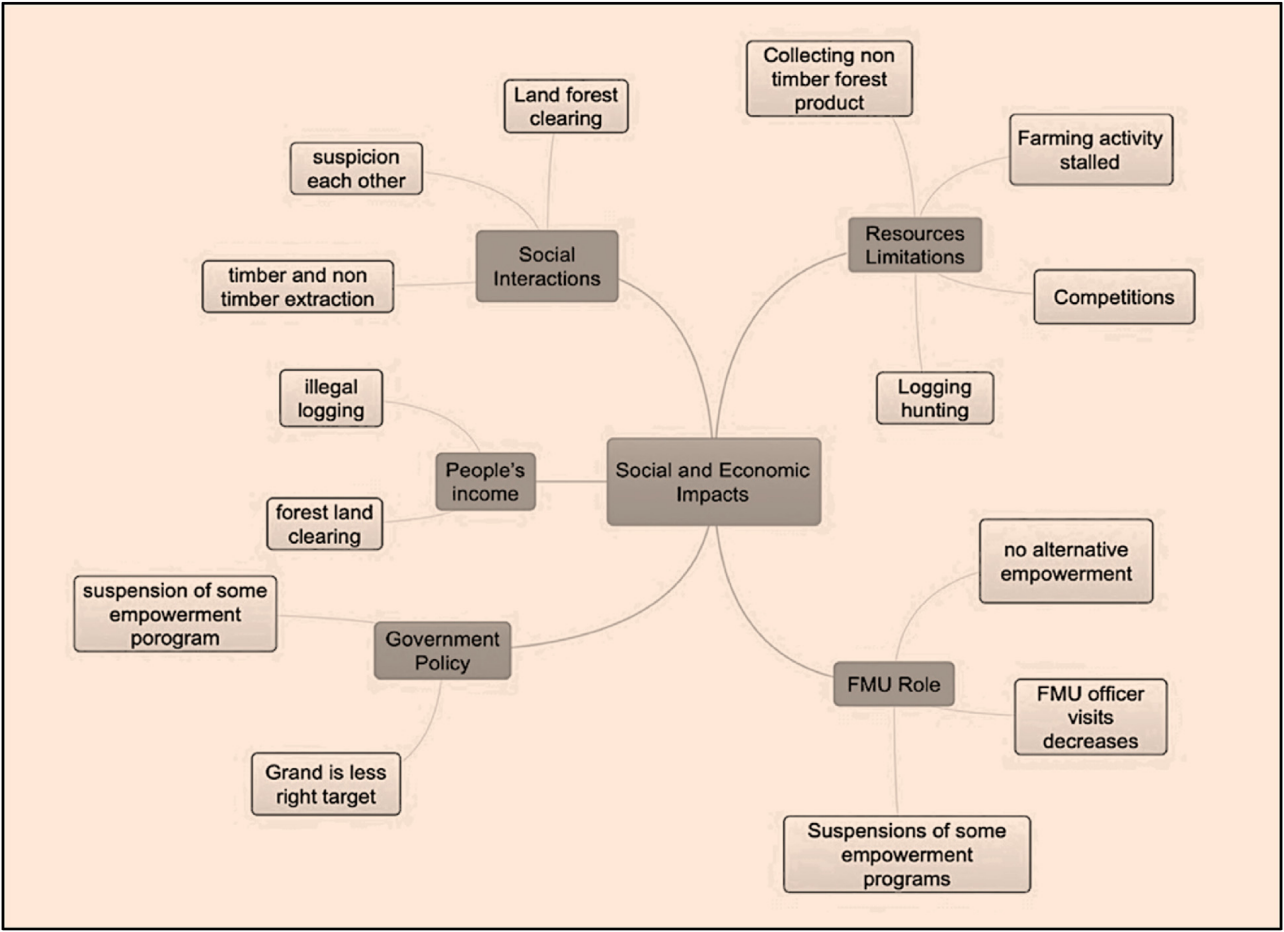
Mind mapping of the social and economic impacts in the FMU's region.

According to the data in Table 3, mind mapping identifies five threats to the communities surrounded by the forest: *social interactions; people's income, limited resources; FMUs role; and Government policy* (see Figure 4).

In some areas, the FMU community is very concerned about being infected by COVID-19. It causes them to be very cautious when everyone comes to the villages. Coupled with everyone fears the potential of transmitting the virus. For this reason, the town issued rules to restrict anyone coming to the city. Some social and economic activities are also limited. Worshiping venues have not been used for a while; schools have been closed, government and private officers must perform Work From Home (WFH). It is consequently "*reduce social interaction*" in the village.

Economically, the restrictions have made a broad impact on the community's economy. For those who do not have a steady job, these social restrictions will cause job losses and directly "*reduce their income*." Even though the government provides direct cash assistance (BLT) for those directly affected due to the pandemic, they still think it's not enough to fulfill their needs. This condition causes "*the community encourages to look for alternative income*". It will generate a new problem in the village that the unemployed citizen will be "*a new competitor*," utilizing natural resources.

**Figure 5:**
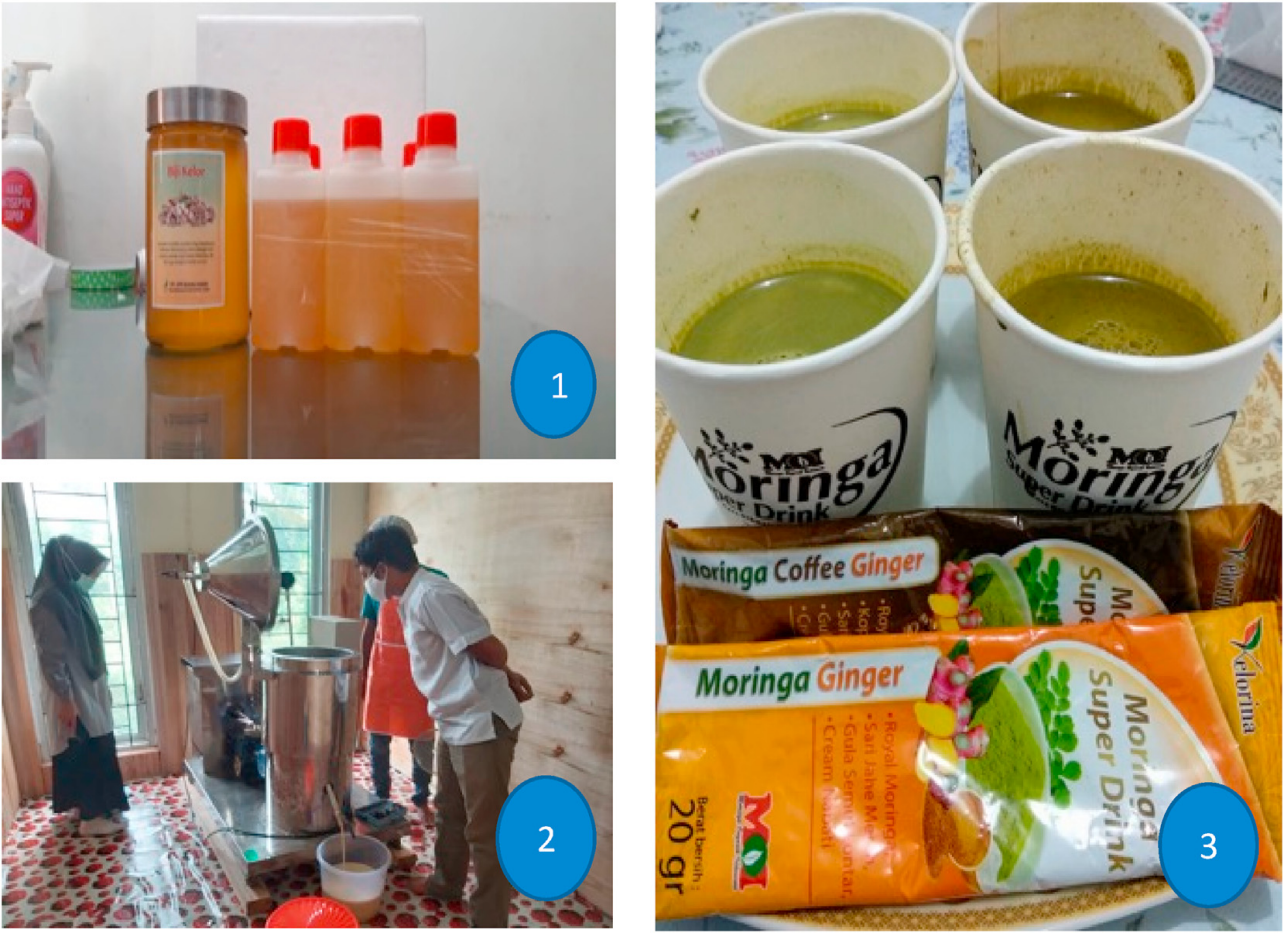
Non-timber processing products in FMU region: (1) Honey bee home production in FMU Kulawi; (2) The processing of natural honey bee in FMU Sintuwu Maroso; (3) The product of Kelor leaf extract (Moringa) in Banawa Lalundu *(Source: Forestry service of Central Sulawesi province 2020).*

Besides that, the "*temporary stop of empowerment programs*" by the FMU has impacted the short-term and long-term consequences community's income (see Figure 5). The group's production unit became hampered due to the reduced FMU's activities in the village. Coupled with the mentoring program stopped temporarily too, caused the community does not know what to do.

Studies conducted by [19]; and [20] explained that whenever the urgency of need exceeds the capacity of resources, people tend to seek other alternatives. They utilized the forest resources by harvesting timber and non-timber forest products instantly, even though it is the state forest [12]. These forest products are for on-farm consumption and market sale in the pandemic situation [6, 17, 21, 22].

#### 3.2.2 Impact of COVID-19 on deforestation and land degradation

Cannot identify the pandemic impact on deforestation and land degradation quickly [9]. This article is only the potential deforestation and land-use changes due to forest resources utilization in state forests. As explained before, society's perceived impact due to the implemented restrictions makes it difficult for them to earn income [23]. Those with useful resources, such as landowners and other economic gain sources, can still survive [7]. However, those who have no properties and only expect to live as farm workers or forest pickers will feel a more significant impact.

Before the pandemic, communities were active in forest resource utilization activities facilitated by FMU. Forest utilization consists of timber and non-timber products, environmental services, and land use is shown in Table 4. Through the facilities of FMU, communities have access to utilize the forest following the characteristics and functions of the region [24]. Economically, the program is beneficial to the rural communities around the forest.

**Figure 6:**
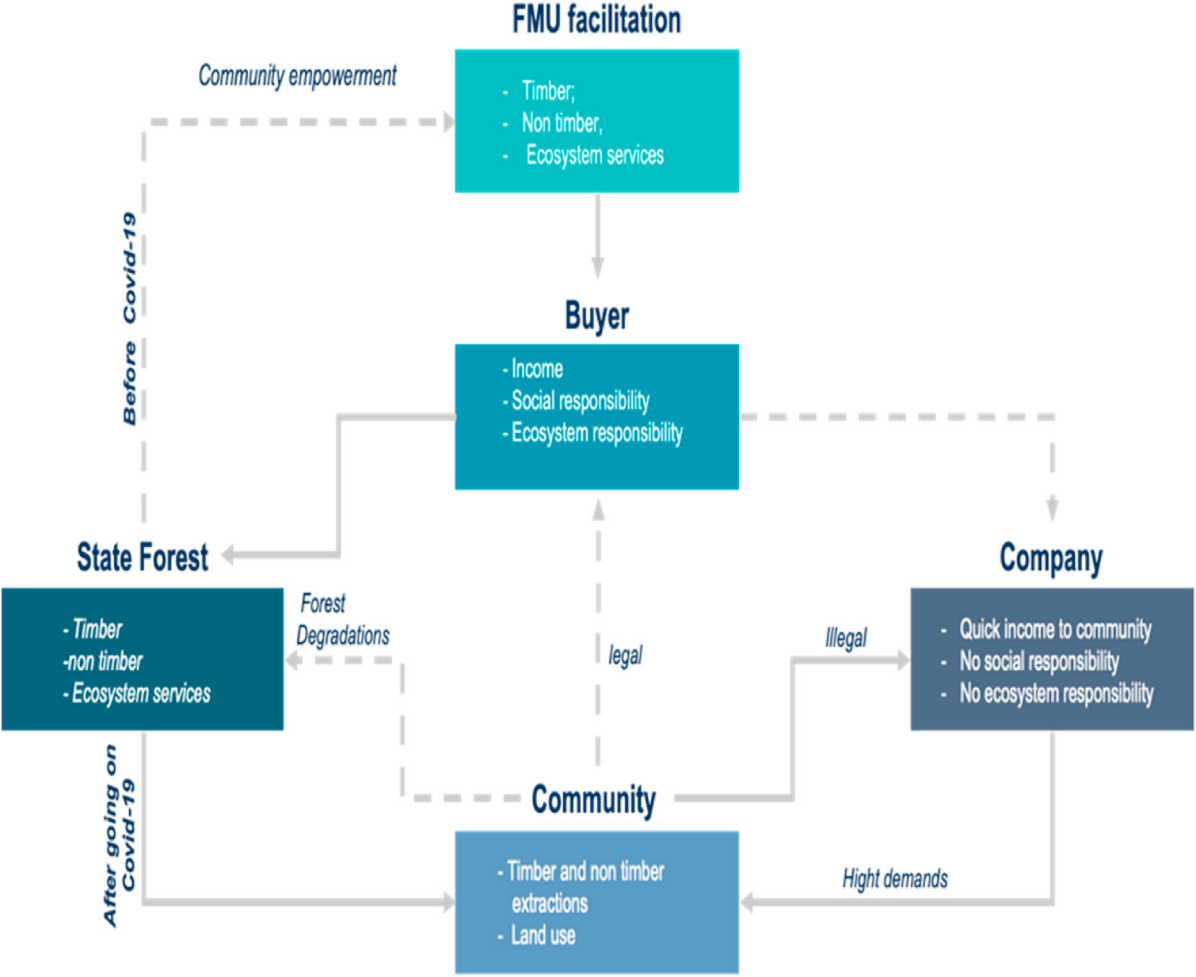
Forest resources used by communities in the FMU region (before and after the COVID-19 pandemic).

Currently, all FMUs activities were suspended, likewise community assistance in the field. That is to say, this situation raises uncertainty for the farmer groups and villagers. There is various speculation to find alternative work that can cover their families' economic needs, such as forest land utilization. Research conducted by [11] explained that while rural communities around the forest face the insistence on economic needs, the forest resources become one of the most accessible alternatives to earning their income (see Figure 6).

The highest-rated forest resources are timber products [25] and other non-timber forest products such as rattan, resin, and palm sugar [22]. In this situation, the communities desperately need to perform activities that provide income to be gained in a short time [26]. They prefer to cut trees for the high demand from the illegal buyer [27].

**Figure 7:**
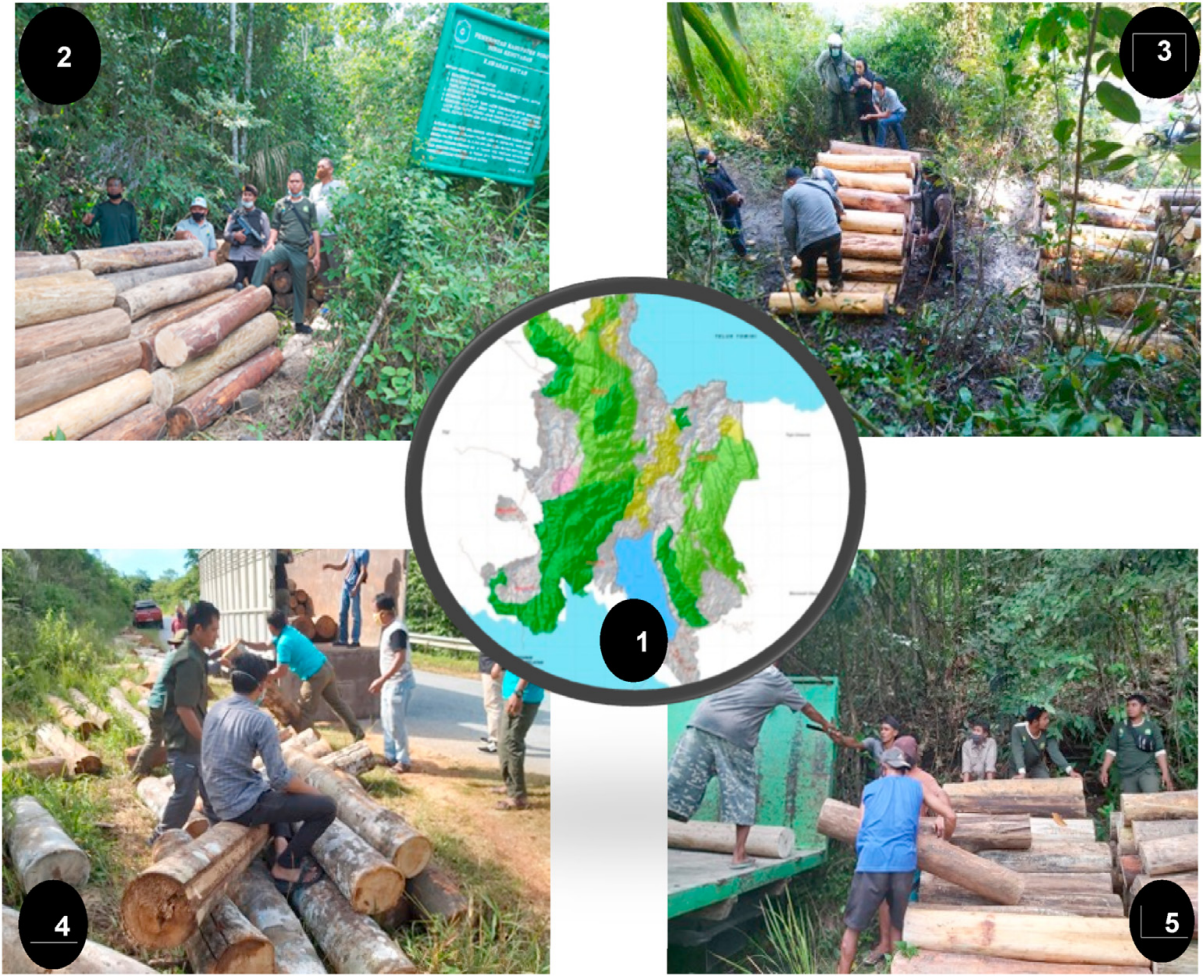
The timber of illegal logging: (1) The territory of FMU Sintuwu Maroso; (2) & (3) The log founded in the state forest area; (4) & (5) Timber located on the roadside *(Source: FMU Sintuwu Maroso, 18 Mei 2020).*

The FMU Sintuwu Maroso and Dampelas Tinombo have found illegal logging activities conducted by the villagers who live near the forest. They take advantage of the opportunity during the COVID-19 situation, where forest patrol activities do not run typically. A fortune that the forest ranger officers managed to arrest him (Figure 7). The results of the investigation conducted by the Forest Ranger are known to some reasons why they do that:

1. The needs of family dining during the pandemic, causing to timber harvesting;
2. They claimed that do not understand the regulation related to the mechanism of licensing of the timber utilizing on state's forest;
3. To supply the High demand from the illegal buyer.

These three reasons are dilemmas. Meanwhile, there is no other choice but to do timber harvesting in the forest [28]. According to [29] the rejection of government policy on forest management is due to the absence of other alternatives that can be used to compensate for forest logging people. Likewise [30] and [13] confirmed that the advanced factor, due to the weak control of maintainers at the field level, was the impetus to the emergence of illegal logging activities in forested areas.

The strict action taken by the FMU Sintuwu Maroso is an effort to preserve the forest's sustainability [24, 31, 32]. However, this is not acceptable by the parties concerned with the timber product. There will be resistance from the associated persons due to the insistence on economic needs. The form of resistance that is often expressed is by logging elsewhere, which is not tracked by the officers [33].

#### 3.2.3 Restore the social and economic condition

Base on the strategic roles of FMU is: optimizing people's access to forests being one of the ways for conflict resolution and optimizing potential timber, non-timber, and environmental services. FMU has a responsibility to help communities face the economic crisis caused by the pandemic, in the short-term and long-term.

Furthermore, FMUs must immediately reactivate empowerment programs that have stopped during the pandemic—prioritized on optimizing the utilization of non-timber forest products and environmental services [34, 35, 36, 37]. The form of forest utilization that can be done has a low risk of the potential spread of COVID-19 and quickly generates money [7, 21, 38]. That is to say, it must do both individually and small groups such as the utilization of resin sap, honey bee cultivation, brown sugar, and environmental services. This activity can provide optimal results when managed professionally and accompanied by FMU to its marketing [39, 40, 41].

## 4 Conclusion

The government policy to prevent the transmission of COVID-19, which affects both the social and economic aspects of society, is inevitable. The available forest resources are the main alternatives for the communities around the forest that have no option to maintain their families' survival.

The utilization of the timber forest and land use will impact the forest's sustainability, directly or indirectly. The prevention efforts undertaken by the FMU by limiting access to forest areas are not the best alternatives during the COVID-19 pandemic. Government policy and quick response from FMU's managers are required. Some creative programs of FMU are needed to implement the COVID-19 countermeasures in an economic improvement program and can provide the income immediately to the communities. It has to be mentored in implementing the program from the early stages until it earns the income.

Motivation and confidence have been given to communities through the program. It has to be prepared by the government and directly coordinated to FMU. It will be one of the best alternatives in overcoming the impact of the COVID-19 in the social and economic aspects. Besides, the active role of FMU in monitoring activities against the threat of forest encroachment should be increased so that it can minimize the damaging impact.

## Declarations

### Author contribution statement

Golar Golar, Hasriani Muis: Conceived and designed the experiments; Performed the experiments; Analyzed and interpreted the data; Wrote the paper.

Adam Malik: Conceived and designed the experiments; Analyzed and interpreted the data.

Achmad Herman: Analyzed and interpreted the data; Contributed reagents, materials, analysis tools or data.

Nurudin Nurudin, Lukman Lukman: Contributed reagents, materials, analysis tools or data.

### Funding statement

This work was supported by the Research Incentives in The Professor Acceleration Program, 10.13039/501100010602Tadulako University 2020.

### Declaration of interests statement

The authors declare no conflict of interest.

### Additional information

The questionnaire associated with this study has been deposited in Google Forms at https://forms.gle/ngUGP3omUpxEr87w7.
